# Platelet Derived Vesicles Enhance the TGF-beta Signaling Pathway of M1 Macrophage

**DOI:** 10.3389/fendo.2022.868893

**Published:** 2022-03-18

**Authors:** Nan Song, Kaifeng Pan, Lei Chen, Keke Jin

**Affiliations:** ^1^Department of Pathophysiology, Wenzhou Medical University, Wenzhou, China; ^2^Key Laboratory of Intelligent Critical Care and Life Support Research of Zhejiang Province, Wenzhou, China; ^3^Zhejiang Decell Biotechnology Co. LTD, Hangzhou, China; ^4^Department of Orthopaedics, The First Affiliated Hospital of Wenzhou Medical University, Wenzhou, China

**Keywords:** nanovesicles, inflammation, immune modulation, platelets, macrophages

## Abstract

Macrophages, mainly divided into M1 pro-inflammatory and M2 anti-inflammatory types, play a key role in the transition from inflammation to repair after trauma. In chronic inflammation, such as diabetes and complex bone injury, or the process of certain inflammatory specific emergencies, the ratio of M1/M2 cell populations is imbalanced so that M1-macrophages cannot be converted into M2 macrophages in time, resulting in delayed trauma repair. Early and timely transformation of macrophages from the pro-inflammatory M1-type into the pro-reparative M2-type is an effective strategy to guide trauma repair and establish the original homeostasis. We prepared purified nano-platelet vesicles (NPVs) and assessed their effects on macrophage phenotype switching through transcriptome analysis. The results elucidate that NPVs promote pathways related to angiogenesis, collagen synthesis, cell adhesion, and migration in macrophages, and we speculate that these advantages may promote healing in traumatic diseases.

## 1 Introduction

Macrophages play a key role in the transition from inflammation to repair after trauma, and they are mainly divided into M1 pro-inflammatory and M2 anti-inflammatory (1). Briefly, 2–3 days after injury, circulating blood monocytes are recruited to the injured tissue and converted into type 1 activated pro-inflammatory macrophages under the stimulation of cytokines, such as TNF-α and IL-17, promoting the inflammatory burst and tissue destruction. At a later stage, monocyte-macrophages produce transforming growth factor-beta (TGF-β), interleukin 10 (IL-10), and other mediators to switch to the M2 phenotype and promote tissue repair. Polarization of macrophages from M1 to M2 reduced inflammation and promoted tissue repair and regeneration. In some chronic inflammations or specific acute inflammatory processes, the high ratio of M1/M2 cell populations and excessive activation of M1 cell populations can lead to severe tissue damage, uncontrolled disease development, and trigger a severe storm of inflammatory factors. It can be speculated that the timely transformation of macrophages from the pro-inflammatory M1-type into the pro-reparative M2-type in the early stage of inflammation is an effective strategy to guide tissue repair and establish the original homeostasis.

Platelets have received increasing attention in the field of regenerative medicine because activated platelets can sustainably release large amounts of growth factors and cytokines, such as platelet-derived growth factor (PDGF), TGF-β, and vascular endothelial growth factor (VEGF), which play an important role in tissue healing and regeneration (2). However, platelets are key effectors in many inflammatory diseases and can promote inflammatory and immune processes by releasing inflammatory cytokines. Therefore, it is theoretically inadvisable to directly apply platelets to diseases in which the M1–M2 transition is deregulated. Meanwhile, extracellular vesicles have gradually attracted attention as a new treatment modality. Recent research suggests that extracellular vesicles in many cell types confer new hope to tissue repair processes (3). Unlike platelets, the role of platelet vesicles on inflammation does not seem to have a unified understanding and seems to have dual pro-inflammatory and anti-inflammatory effects (4), these different effects may be due to the different preparation methods of vesicles. TGF-β is a multifunctional cytokine that maintains skin homeostasis, mainly produced by platelet activation, and can stimulate collagen synthesis, fibroblast proliferation, and angiogenesis after trauma (5). It has been reported in the literature that TGF-β can transform M1 macrophages into M2 cells and accelerate angiogenesis and tissue repair (6–10).

Herein, we prepared purified NPVs and evaluated their structure and component retention. The results showed that NPVs are nanoscale platelet vesicles that can produce large amounts of TGF-β upon activation. Then, its regulatory effect on the macrophage phenotype was investigated using transcriptomics. The results of Gene Ontology (GO) enrichment analysis depicted that NPVs promoted angiogenesis, cell migration, cell adhesion, and collagen synthesis. Gene set enrichment analysis (GSEA) results further elucidated that NPVs could activate the TGF-β signal transduction pathway in macrophages and its related genes (Bmp4/Smad9/Inhbb/Tgfb3/Thbs3/Thbs2/Bmp6) were significantly upregulated, which could promote angiogenesis, cell adhesion, and migration, contributing to wound repair. Therefore, NPVs can promote the healing of traumatic diseases by regulating the TGF-β pathway.

## 2 Materials and Methods

### 2.1 Materials

Transforming growth factor-beta (TGF-β) human ELISA kit were purchased from Thermofisher Scientific. The antibodies for western blotting were TSG101 Polyclonal Antibody (28283-1-AP, dilution: 1:2000) and CD41/Integrin Alpha 2B Polyclonal Antibody (24552-1-AP, dilution: 1:1000). The antibody for immunofluorescence was TGF Beta 2-Specific Polyclonal Antibody (19999-1-AP, dilution: 1:200) from Proteintech. Prostaglandin E1(PGE1) was purchased from Sigma-Aldrich.

### 2.2 Preparation of NPVs

Platelets were isolated from the blood of volunteers according to reported protocols (11). The whole sample of fresh blood was centrifuged at room temperature at 100 × g for 20 min. After centrifugation, the whole blood sample was divided into three layers, from top to bottom: the plasma layer, white blood cell layer, and red blood cell layer. The pale-yellow plasma layer was collected and centrifuged at 800 × g for 20 min. After centrifugation, the pellets were platelets. The pellet was resuspended in 1 μM PGE1 in phosphate buffered saline to inhibit platelet aggregation and stored at room temperature until use. The platelet solution of 1 × 10^6^ cells/mL was squeezed six times with 400nm, 200nm, and 100nm pore size polycarbonate membrane filters (Whatman) in a micro extruder (Avanti Polar Lipids), and NPVs were obtained after centrifugation.

### 2.3 Characterization of NPVs

#### 2.3.1 Dynamic Light Scattering (DLS) and Cryo-Transmission Electron Microscopy (Cryo-TEM)

NPV size was measured by DLS using a Malvern Zetasizer Nano ZS. The morphology of the NPVs was observed using a cryo-transmission electron microscope (200 kV, FEI Tecnai G2 F20, USA). Briefly, 2 μl of the sample solution was applied to a Cryo-TEM Cu grid (R1.2/1.3 Quantifoil Jena grid), which was held by tweezers inside a controlled environment vitrification system. The perforated films exhibited good carrier wettability by glow-discharge air plasma cleaning. Immediately after the droplets were placed on the grid and blotted dry, the grid was quickly plunged into liquid ethane with a freezing point of -183°C. Cryo-TEM imaging was performed at 3 μm focusing on an FEI-Talos F200C TEM equipped with a field emission gun operating at 200 kV. Images were recorded using a 4k × 4k Ceta camera.

#### 2.3.2 Western Blot Analysis

Platelets and NPVs were directly lysed using radioimmunoprecipitation assay buffering. Twenty micrograms of protein were separated by 10% SDS-PAGE electrophoresis, and the protein was blotted onto a polyvinyl fluoride membrane. The cells were blocked with 10% serum in FBS (pH 7.4) for 1 h and incubated with primary antibody overnight at 4°C. After three washes with TBST, the cells were co-incubated with secondary antibodies at a 1:1000 dilution for 2 h. Finally, images were acquired using the Amersham Imager 600 imaging system (General Electric Company, USA).

#### 2.3.3 TGF-β Release Study

Platelets and NPVs were placed in 12-well plates, with or without 5% calcium chloride solution per sample, and after 2 h incubation in a CO_2_ incubator at 37°C, the samples were taken out and centrifuged at 16,000 g at 4°C for 20 min. TGF-β concentrations were determined using an enzyme linked immunosorbent assay (ELISA) kit according to the manufacturer’s protocol.

#### 2.3.4 Extraction of Mouse Bone Marrow-Derived Macrophages (BMDMs)

BMDMs are widely used as primary macrophages that are differentiated from mammalian bone marrow cells. Briefly, 6–8-week-old male C57BL/6 mice were euthanized by cervical dislocation after anesthesia. The femur and tibia were removed, both ends cut off, and the medium was pushed into the bone with 1 mL syringe to extract the bone marrow, added 10% heat-inactivated fetal bovine serum was used, 1% penicillin-streptomycin, and M-CSF, and differentiated and cultured the bone marrow in RPMI 1640 medium. After 7 days, the attached BMDMs were collected and used for further experiments.

#### 2.3.5 Immunofluorescent Analysis

The cells in the plate were gently washed twice with PBS, fixed in 4% paraformaldehyde for 15 min, placed in 0.5% v/v Triton X-100 solution for 10 min, and then blocked with 10% goat serum for non-specific binding protein. It was then incubated with the primary antibody (TGF Beta 2-Specific Polyclonal Antibody) at 37°C for 90 minutes. After 3 washes with PBS, each group was incubated with FITC-conjugated goat anti-rabbit IgG for 90 min at room temperature and washed three times with PBS. Afterwards, the DAPI working solution was incubated for 15 min. After washing, the slides were mounted and observed with a LEICA DMI8 laser confocal microscope (Leica, Heidelberg, Germany).

### 2.4 Transcriptomic Study

BMDMs were stimulated with 500 ng/mL LPS for 6 h to induce the M1 macrophages. Then, PBS or 100 μg/mL NPVs were added, and total RNA triplets were isolated using the TruSeq RNA Sample Prep Kit (Illumina, San Diego, CA, USA). RNA-sequencing analysis was performed using individual biotechnology to generate 150 bp paired-end reads. Reference genomes and annotation files were downloaded from the ENSEMBL database (http://www.ensembl.org/index.html). GO enrichment analysis and GSEA were performed using the OmicStudio tool of the Lianchuan Biocloud Platform (https://www.omstudio.cn/tool.). Genes with a P-value < 0.05 and an absolute log2-fold change ≥ 1 were identified as differentially expressed genes. The degree of enrichment and statistical significance were quantified using the normalized enrichment score and false discovery rate.

### 2.5 Statistical Analysis

Statistical significance between two groups was analyzed by a t-test using GraphPad Prism software (No. 8, San Diego, USA). Data are presented as mean ± SEM. The correlation between two parameters was assessed using Spearman’s correlation analysis. Statistical significance was set at p < 0.05.

## 3 Results and Discussion

### 3.1 Characterization of NPVs

**Figure 1:**
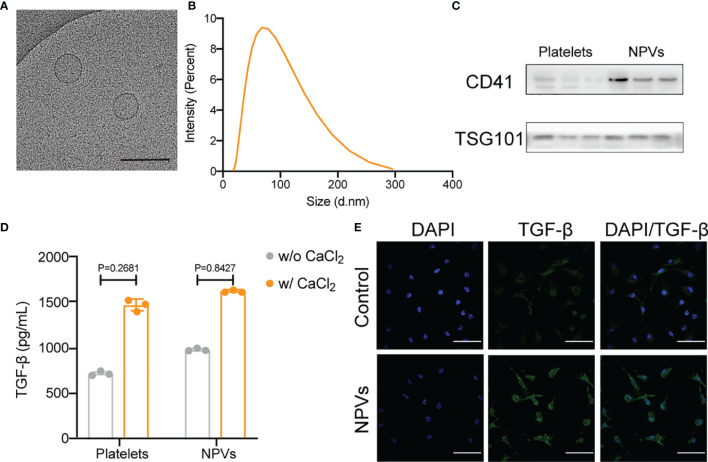
Characterization of NPVs. **(A)** Representative cryo-TEM images of NPVs. (Scale bar; 1 bar represents 200 nm) **(B)** Size distribution of NPVs obtained using DLS, NPVs are on average about 68 nm wide. **(C)** The expression of CD41 on NPVs was detected by western blot, and TSG101 was used as an internal reference. **(D)** ELISA detection of TGF-β content in platelets and NPVs. **(E)** Immunostaining of M1 macrophages after treatment with phosphate buffered saline (PBS) or NPVs. (Nuclei, blue; TGF-β, green) Scale bar = 100 μm, n = 3 per group.

We purified and isolated platelets from fresh human platelet-rich plasma and obtained NPVs by extrusion. The purified NPVs were characterized by cryo-transmission electron microscopy (cryo-TEM), dynamic light scattering analysis (DLS), western blotting, and ELISA. We measured the size of the vesicle using DLS and found it to be approximately 68 nm in diameter (**Figure 1A**). To determine the ultrastructure of NPVs, we visualized the purified NPVs in PBS buffer using cryo-TEM (**Figure 1B**). The results show that NPVs are membrane-coated vesicles with a diameter of less than 200 nm, which is consistent with that observed with DLS. The results of the western blotting analysis showed that the platelet marker CD41 was also enriched in the NPVs, proving that the NPVs were derived from platelets (**Figure 1C**). Platelets promote tissue healing and regeneration by releasing growth factors after activation. To investigate whether NPVs also have this property, we activated platelets and NPVs with calcium chloride solution, and then detected transforming growth factor-beta (TGF-β) in the supernatant by ELISA (**Figure 1D**). Like platelets, NPVs significantly release TGF-β upon calcium chloride activation. To verify the role of NPVs on inflammatory cells, we extracted BMDMs (primary macrophages) from mouse femur and tibia, stimulated them with LPS for 6 h to differentiate into M1 macrophages. After incubation with PBS or NPVs for 4 h, the fluorescence intensity of TGF-β in M1 macrophages was observed by confocal microscope (**Figure 1E**). Compared with the control group, the fluorescence intensity of TGF-β in M1 macrophages was significantly enhanced after adding NPVs. This indicates that NPVs can play a role by increasing the content of TGF-β in M1 macrophages. These experimental results demonstrate that we successfully prepared nanoscale vesicles that retain the essential characteristics of platelets and were enriched with TGF-β.

### 3.2 Transcriptomic Study of Macrophages Treated With NPVs

**Figure 2:**
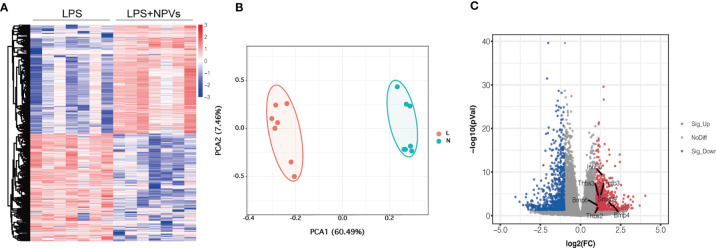
Transcriptomic studies of macrophages treated with NPVs. **(A)** Differentially expressed genes in the transcriptomes of LPS group (left) and LPS+NPVs group (right) (n = 7, the screened differential genes were consistent with |log2FC| > 0.5, p < 0.05). **(B)** PCA analysis depicted that the LPS group (L) and the LPS+NPVs group (N) were significantly different in space. **(C)** Volcano plot showing upregulation of TGF-β pathway-related genes after NPS-treated macrophages. (Marked genes are significantly different, p < 0.05).

Encouraged by previous experimental data, we investigated whether macrophage polarization could be regulated by NPVs. We added NPVs to M1 macrophages to study their effect on macrophages by transcriptome analysis. The heatmap depicts that a total of 27,271 genes were detected in the two groups, and 1,180 differentially expressed genes were screened according to absolute log2 fold change ≥ 1 and P-value < 0.05 (**Figure 2A**). Compared with the LPS group, 594 genes were upregulated in the LPS+NPVs group, and 586 genes were downregulated. PCA revealed that the genes in the LPS and LPS + NPVs groups were spatially distinct (**Figure 2B**). Then, we wanted to determine whether TGF-β was associated with significant differences between the two groups; therefore, we analyzed the genes of the TGF-β pathway. Volcano plots depicted upregulation of TGF-β pathway-related Bmp4/Smad9/Inhbb/Tgfb3/Thbs3/Thbs2/Bmp6 gene expression (**Figure 2C**).

### 3.3 Gene Ontology (GO) Enrichment Analysis

**Figure 3:**
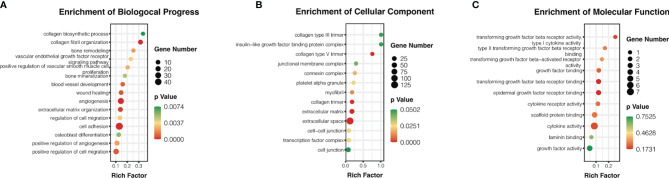
The potential biological functions of NPVs were analyzed by Gene Ontology (GO). **(A)** is biological process analysis, **(B)** is cell component analysis, and **(C)** is molecular function analysis. The pathways which are shown in the figure all help promote tissue repair. Biological processes are listed in the figure with their respective gene numbers and p-values.

The potential biological functions of the NPVs were analyzed using GO enrichment analysis. The results showed that the differentially expressed genes were mainly enriched in biological processes and cellular components. Bioprocess analysis showed that NPVs promoted angiogenesis, cell migration, cell adhesion, and collagen synthesis (**Figure 3A**). Further analysis of cellular components revealed that macrophages stimulated by NPVs contained more collagen trimers, transcription factor complexes, and connexin complexes, which have potential positive regulatory functions for inducing M2 polarization (**Figure 3B**). Molecular functional analysis indicated that these components may be involved in the binding of transforming growth factor-beta receptor, epidermal growth factor receptor, and laminin, which contribute to tissue repair (**Figure 3C**).

### 3.4 Gene Set Enrichment Analysis (GSEA)

**Figure 4:**
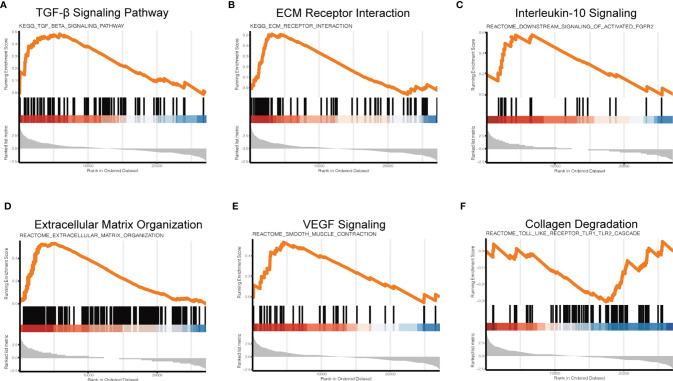
Gene set enrichment analysis (GSEA) to investigate changes in signaling pathways in macrophages after NPVs treatment. **(A)** is the TGF-β signaling pathway, **(B)** is ECM receptor interaction, **(C)** is IL-10 signaling, **(D)** is extracellular matrix organization, **(E)** is VEGF signaling, and **(F)** is collagen degradation.

To further investigate the regulatory mechanism of NPVs on macrophage signaling pathways, we used GSEA to investigate the changes in macrophage signaling pathways. Compared with the LPS group, the LPS + NPVs group depicted enrichment scores for the TGF-β signaling pathway (**Figure 4A**), ECM receptor interaction (**Figure 4B**), IL-10 signaling (**Figure 4C**), extracellular matrix organization (**Figure 4D**), and VEGF signaling (**Figure 4E**) were 0.48, 0.51, 0.58, 0.26, and 0.53, respectively, indicating that these genes were upregulated, while collagen degradation (**Figure 4F**) had an enrichment score of -0.31, indicating that their gene expression was downregulated. In an inflammatory environment, these specific groups of genes (TGF-β, IL-10, VEGF) can drive the M1-M2 transition of macrophages, inhibit abnormal inflammatory responses, promote angiogenesis, promote the formation of collagen tissue, and inhibit collagen degradation. Therefore, we believe that NPVs have potential pro-reparative effects and can be applied to various diseases with a dysregulated m1-m2 transition to promote tissue repair and regeneration.

Under normal circumstances, repair after tissue damage is rapid and efficient. However, in chronic non-healing injury (such as diabetic ulcers and other diseases), the local injury site cannot enter the repair stage, resulting in impaired vascularization and delayed repair process (12).

Macrophages play a key role in injury repair. Not only participating in different stages of the healing response but also switching phenotypes in time has a profound impact on specific repair mechanisms. Meanwhile, it has been reported that impairment of TGF-β signaling and defects in inflammation resolution promotes delayed healing leading to injury (13). Here, we prepared NPVs and demonstrated at the transcriptomic level that they could upregulate some genes of the TGF-β pathway, which can help tissue repair and regeneration. These include the bone morphogenetic proteins BMP4 and BMP6, which are multifunctional growth factors whose high expression is associated with pro-reparative macrophage differentiation (14). Bmp is involved in a variety of physiological processes, such as regulation of angiogenesis, cell migration adhesion, and collagen formation (15). In addition, NPVs also upregulated IL-10 signaling. IL-10 is a powerful negative feedback regulator of inflammation, which inhibits pro-inflammatory responses, balances the local inflammatory microenvironment, and induces M2 macrophages to enter the wound healing and pro-repair stage (16)in conclusion, NPVs can elevate the TGF-β pathway in macrophages, resulting in a pro-reparative phenotype.

However, we did not perform an animal model to validate the anti-inflammatory effect of NPV *in vivo*, which should be analyzed in future studies. We believe this may provide new therapeutic strategies and new insights for various diseases with dysregulated M1–M2 transitions.

## 4 Conclusion

In conclusion, we prepared nanoscale platelet vesicles from top to bottom, which can alter the function of M1-type macrophages through the TGF-β pathway promoting the interaction of angiogenesis, collagen synthesis, cell adhesion, and migration-related pathways. we speculate that these advantages of nanoscale platelet vesicles may promote the healing of traumatic diseases.

## Data Availability Statement

The datasets presented in this study can be found in online repositories. The names of the repository/repositories and accession number(s) can be found below: https://www.ncbi.nlm.nih.gov/, PRJNA800616.

## Ethics Statement

The animal study was reviewed and approved by the animal experimental ethics committee of Sir Run Shaw Hospital.

## Author Contributions

KJ and LC contributed to conception and design of the study. KP organized the database. NS performed the statistical analysis and wrote the first draft of the manuscript. All authors contributed to manuscript revision, read, and approved the submitted version

## Funding

The study was supported by the Wenzhou major science and technology project (2018ZY015), the basic scientific research project of Wenzhou Science and Technology Bureau (Grant No. Y2020238) and Basic Research of Wenzhou Science and Technology Bureau (Y2020009). 

## Conflict of Interest

Author NS was employed by Zhejiang Decell Biotechnology Co. LTD.

The remaining authors declare that the research was conducted in the absence of any commercial or financial relationships that could be construed as a potential conflict of interest.

## Publisher’s Note

All claims expressed in this article are solely those of the authors and do not necessarily represent those of their affiliated organizations, or those of the publisher, the editors and the reviewers. Any product that may be evaluated in this article, or claim that may be made by its manufacturer, is not guaranteed or endorsed by the publisher.
